# Games for the Men

**Published:** 1918-03-16

**Authors:** John Penoyre

**Affiliations:** Address for Sending games to: Sir Edward Ward, D.G.V.O., 45 Horseferry Road, S.W. 1. Letters to John Penoyre, 8 King's Bench Walk, Temple, E.C. 4.


### The Editor's Letter-Box.

### Games for the Men.

*To the Editor of* The Hospital.

Sir,—Can you, even in these days, find me a corner to ask, on behalf of Sir Edward Ward, Director-General of Voluntary Organisations, for gifts of games of all kinds for the men? The unlooked-for kindness shown the sweater industry by your readers during the past four years makes me bold to prefer this request.

We are constantly asked at the depôts at Havre, Basra, and elsewhere for games, and one can well believe that they add greatly to the happiness and well-being of those who do so much for us. After all, games are rather a British speciality, and probably most houses could spare one for the men—only we must remember who they are for, and send, as always, our best. Here is a first list of what we want: Everything used for bowls, boxing, cards, chess, cricket, croquet, draughts, football, golf, Halma, "House," and similar games, hockey, quoits, and tennis. Also the kit needed, especially shoes and shorts. Some of the above are, of course, for convalescents. Any addition dictated by good sense and good feeling, rather than spring-cleaning, can be made to this list.

Kindly enclose name and address in parcels.—Yours faithfully,

John Penoyre.

Address for sending games to:
Sir Edward Ward, D.G.V.O.,
45 Horseferry Road, S.W. 1.

Letters to John Penoyre,
8 King's Bench Walk, Temple, E.C. 4.
March 6, 1918.

